# Lipase-Catalyzed Synthesis of Sugar Esters in Honey and Agave Syrup

**DOI:** 10.3389/fchem.2018.00024

**Published:** 2018-02-12

**Authors:** Sascha Siebenhaller, Julian Gentes, Alba Infantes, Claudia Muhle-Goll, Frank Kirschhöfer, Gerald Brenner-Weiß, Katrin Ochsenreither, Christoph Syldatk

**Affiliations:** ^1^Technical Biology, Institute of Process Engineering in Life Sciences, Karlsruhe Institute of Technology, Karlsruhe, Germany; ^2^Institute of Organic Chemistry and Institute for Biological Interfaces 4, Karlsruhe Institute of Technology, Karlsruhe, Germany; ^3^Bioengineering and Biosystems, Institute of Functional Interfaces, Karlsruhe Institute of Technology, Karlsruhe, Germany

**Keywords:** sugar ester, glycolipid synthesis, honey, agave syrup, lipase, transesterification, deep eutectic solvent

## Abstract

Honey and agave syrup are high quality natural products and consist of more than 80% sugars. They are used as sweeteners, and are ingredients of cosmetics or medical ointments. Furthermore, both have low water content, are often liquid at room temperature and resemble some known sugar-based deep eutectic solvents (DES). Since it has been shown that it is possible to synthesize sugar esters in these DESs, in the current work honey or, as vegan alternative, agave syrup are used simultaneously as solvent and substrate for the enzymatic sugar ester production. For this purpose, important characteristics of the herein used honey and agave syrup were determined and compared with other available types. Subsequently, an enzymatic transesterification of four fatty acid vinyl esters was accomplished in ordinary honey and agave syrup. Notwithstanding of the high water content for transesterification reactions of the solvent, the successful sugar ester formation was proved by thin-layer chromatography (TLC) and compared to a sugar ester which was synthesized in a conventional DES. For a clear verification of the sugar esters, mass determinations by ESI-Q-ToF experiments and a NMR analysis were done. These environmentally friendly produced sugar esters have the potential to be used in cosmetics or pharmaceuticals, or to enhance their effectiveness.

## Introduction

Many innovations are being developed today by improving and combining existing products or processes or by the usage of alternative substrates. As an example, glycolipids, which are sugar fatty acid esters, had been produced chemically for decades. Nowadays, they are often produced more sustainably by fermentation or synthesized by enzymes from renewable resources (Ducret et al., 1996). Glycolipids are important commercial molecules and their hydrophilic–lipophilic balance (HLB) covers a wide range, depending on the used carbon chain and sugar moiety. Sugar esters are surface active and have emulsifying properties, which make them accessible for countless applications in fine chemicals, pharmaceuticals, and food (Yan et al., 1999). Moreover, and especially for cosmetic applications, glycolipids are also highly interesting. In addition of their capability of cleaning the skin, glycolipids have good physico-chemical properties, have biological activities and a low toxicity, are biodegradable, odor- and tasteless and non-irritant (Coulon et al., 1996; Cao et al., 1999; Degn et al., 1999; Tarahomjoo and Alemzadeh, 2003). Glycolipids are used particularly in toothpaste, lotions, shampoos, and lipsticks (Šabeder et al., 2006), but they have the potential to be used in even more products. An example for their bioactivity is the use of sugar esters as antibacterial agent. In this case, fructose laurate exhibited high growth inhibitory effects against various pathogenic bacterial strains (Watanabe et al., 2000; Šabeder et al., 2006).

By contrast, honey is used since ancient times in dermatology and skin care and is still today an important and often used ingredient for cosmetics and some medical ointments. Honey is composed, depending of the pollen source, climate, environmental conditions, and its processing, of over 180 substances (Gheldof et al., 2002; Azeredo et al., 2003). The main constituents of mixed floral honey are sugars, with an average composition of 40.9% fructose, 35.7% glucose, 1.4% maltose, 0.9% sucrose, 0.2% higher sugars, and 17.1% water (United States Department of Agriculture, 2016). Additionally, other undetermined substances like phenolic acids, flavonoids, proteins and enzymes, amino acids and minerals make up to ~3.2% (White and Doner, 1980). Ohmenhaeuser et al. described in 2013, that the average ratio is 56% fructose to 44% glucose (Ohmenhaeuser et al., 2013). Due to its composition, honey has several functions such as antioxidant, antibacterial, antitumoral, anti-inflammatory, antiviral, and anti-browning agent (Viuda-Martos et al., 2008; Burlando and Cornara, 2013). Furthermore, it has a hydrating effect, keeping the skin juvenile and retarding wrinkle formation (Crane, 1980; Jiménez et al., 1994).

Agave syrup is the extract from the cores of *Agave americana* and *Agave tequilana*. After filtration and heating, the polysaccharides break into monosaccharides. The resulting highly sugary agave syrup is not used in the cosmetic industries, but is often used as sweetener or as a modern and vegan alternative for honey. The sugar composition of agave syrup is similar to honey, with 84.3% fructose, 8.3% glucose, and smaller amounts of saccharose, mannitol, and inositol (Willems and Low, 2012).

In the last decades enzymatic synthesis in non-aqueous media has gotten more and more attention. Due to the high product specificity of enzymes, which leads to virtually pure products, these reactions can be carried out under eco-friendly conditions, by choosing the right solvent. Therefore, new and more sustainable solvents were invented.

One of this eco-friendly and nearly water-free solvent class are deep eutectic solvents (DES), a mixture of an ammonium- or phosphonium salt and a hydrogen-bond donor. After mixing and heating these components, a fluid occurs, which is often liquid at room temperature. DES are generally classified as environmental friendly, biodegradable, non-toxic, non-flammable, and non-volatile (Liu et al., 2015; Kim et al., 2016).

Recent works by Pöhnlein et al. and Siebenhaller et al. shows that it is possible to use a DES, based on choline chloride and a sugar, to synthesize glycolipids (Pöhnlein et al., 2015; Siebenhaller et al., 2016). In this reaction set-up, the sugar used is simultaneously part of the solvent and substrate.

By working with established high sugar-content DES, this system can be possibly combined and improved by using natural sugary products with low water content as substrate and media for the synthesis of sugar esters.

In the present study, the use of honey and agave syrup as solvent and substrate were investigated. In order to do this, important parameters like pH, sugar and water content were determined. Reactions were performed with immobilized *Candida antarctica* lipase B (iCalB) and different fatty acid vinyl esters as substrate. The resulting products of performed reactions were extracted and analyzed via thin-layer chromatography (TLC), mass spectrometry, and NMR analysis.

The obtained results may lead in future to a further link between the (bio) chemical and the food, pharmaceutical or cosmetics industry in the production of alternative sugar esters or in more bioactive compounds based on natural products.

## Materials and methods

### Materials

Lipase B from *Candida antarctica*, immobilized on acrylic resin (iCalB) and choline chloride (98%) were purchased from Sigma-Aldrich (Germany). Commercially available honey (“Flotte Biene, Obstblütenhonig,” Langnese Honig GmbH & Co., KG, Germany) and agave syrup (“Agaven Dicksaft Fruchtsüße,” dmBio, dm-drogerie markt GmbH + Co., KG, Germany) were used as substrates. All used fatty acid vinyl esters were acquired from Tokyo Chemical Industry Co., Ltd. (TCI-Europe, Belgium). If not stated otherwise, all other chemicals were purchased from Carl-Roth (Germany).

### Methods

#### Enzymatic synthesis of glycolipids

The enzymatic synthesis of glycolipids is based on Siebenhaller et al. with slight modifications as follows: 20 mg of iCalB, 200 μl of a fatty acid vinyl ester (vinyl palmitate, vinyl laurate, vinly decanoate, or vinyl octanoate) and 2.5 ml of honey or agave syrup were filled in a 5 ml Eppendorf Tube (Siebenhaller et al., 2016). After rigorous shaking, the reaction was carried out in a rotator with vortex mixer in program U2 at 50 rpm (neoLab, Germany) at 50°C for 48 h. As a control, reactions without enzyme or without a fatty acid vinyl ester were made.

As a reference, fructose- and glucose-based glycolipids were synthesized in a conventional DES. For this, choline chloride and the corresponding sugar were mixed in a molar ratio of 1:1 at 100°C under constant stirring until a liquid was formed. Afterwards, lipase and fatty acid vinyl ester were added and the reactions were performed as mentioned above.

#### Extraction of glycolipids and purification by flash chromatography

Prior to analysis, synthesized glycolipids were extracted from reaction media by adding 2 ml of warm water and rigorously shaking the obtained mixture. After further addition of 3.5 ml ethyl acetate and shaking for 45 s, a glycolipid-containing organic phase was formed. This was collected and used for TLC and purification.

For purification of synthesis products by flash chromatography (Reveleris Prep, Büchi Labortechnik GmbH, Germany), six identical extracts were unified and concentrated to a volume of ~4 ml. The concentrated phase was applied to a Revelersis HP Silica 12 g column at a flow rate of 30 ml/min, a chloroform:methanol gradient was used for the separation of synthesis products as follows: 0% methanol to 10% in 1.8 min, holding the gradient for 7.1 min. Afterwards, increase to 15% in 3.6 min and to 100% in 1.8 min. It was hold for 1.8 min to remove all sugars. Product peaks were observed by an evaporative light scattering detector (Treshold: 20 mV, Sensitivity: low) and fractionated. Fractions were analyzed via TLC and subsequently used for MS and NMR analysis.

#### Analytical methods

##### Detection of glycolipids via thin-layer chromatography

For qualitative analysis of formed glycolipids, 10 μl of the crude extracts were spotted onto a silica gel TLC plate as stationary phase (Alugram SIL G, 60 Å, Macherey-Nagel GmbH & Co., KG, Germany). The mobile phase consists of chloroform: methanol: acetic acid (65:15:2, by vol.) to separate the synthesized compounds (Pöhnlein et al., 2015). Visualization of the different glycolipids was accomplished by incubation of the TLC plate in a dyeing solution. The dyeing solution consists of anisaldehyde:sulfuric acid:acetic acid (0.5:1:100, by vol.). After incubation, the TLC plate was heated by a 200°C hot air stream for ~5 min.

##### Determination of the accurate masses via electrospray ionization quadrupole time of flight mass spectrometry (ESI-Q-ToF MS)

The accurate masses of the synthesis products, which were separated and purified by flash chromatography, were determined with an ESI-Q-ToF MS system (Q-Star Pulsar i, AB SCIEX, Germany) equipped with an electrospray ionization (ESI) source. All measurements were carried out in the positive mode within a mass range from m/z 50 to m/z 800 using the activated “enhance all” setting. Before measurement, the samples were diluted 1:5 in a mixture of 10 mM ammonium acetate and methanol (1:1, by vol.) and continuously infused via a syringe pump at a flow rate of 10 μl/min.

The ion source voltage was set to 4,800 V, declustering potential to 30 V, and focusing potential to 100 V. As nebulizer and curtain gas, nitrogen gas 5.0 was used in all experiments.

Data acquisition and processing were performed using the Analyst QS 1.1 software (AB SCIEX, Germany).

##### Structural elucidation of sugar esters via nuclear magnetic resonance spectroscopy

For NMR spectroscopy, 12 mg of purified sugar octanoates based on honey and 13 mg based on agave syrup were dissolved in 0.6 ml CDCl3:d6-acetone (4:1, by vol.). 1D ^1^H NMR spectroscopy and 2D ^1^H–^1^H correlation spectroscopy (COSY), ^1^H–^13^C heteronuclear single quantum coherence spectroscopy (clip-HSQC, Enthart et al., 2008), and heteronuclear multiple-bond correlation spectroscopy (HMBC) were recorded with a Bruker AVANCE II +600-MHz spectrometer (Bruker AG, Germany) equipped with a BBI probe head. Recorded spectra were analyzed with Topspin 3.2 (Bruker AG). Intensities were measured from a 1D ^1^H spectrum acquired with a 16 scan and 4 dummy scans. Chemical shifts are referenced to the ^1^H and ^13^C resonance of tetramethylsilan.

#### Characterization of the used honey and agave syrup

The water content of honey and agave syrup was measured via Karl Fischer titration (TitroLine 7500 KF trace, SI Analytics, Germany). Before measuring, the titrator was tested with a water standard (Merck Millipore, Germany).

The water activity a_w_ of honey and agave syrup was determined with an AquaLab CX-2 at 22°C (Decagon Devices, USA).

After calibrating, the pH was directly measured in honey and agave syrup and as a 10% dilution (w/v) (SenTix® Mic, Xylem Analytics, Germany).

The two main carbohydrates, fructose and glucose, were quantified by HPLC (Agilent 1100 Series, Agilent Technology, Germany) with a Rezex ROA organic acid H^+^ (8%) column (300 mm length, 7.8 mm diameter) and a Rezex ROA organic acid H^+^ (8%) guard column (50 mm length, 7.8 mm diameter) from Phenomenex (Phenomenex Ltd., Germany) as described in Dörsam et al. (2017). Separation was performed with 5 mM H_2_SO_4_ for 45 min and a flow rate of 0.5 ml/min under isocratic conditions at 50°C column temperature. Carbohydrates were detected via a refractive index detector (Agilent 1200 series, Agilent Technology, Germany).

Quantification of the sugars were performed by using three different dilutions of honey or agave syrup (0.3–1 mg/ml) and an external 10-point calibration curve for each component from 10 to 500 mg/l.

All measurements were made in triplicates.

## Results

### Characterization of the natural products

For characterizing the used sugar-containing natural products—honey and agave syrup—the contents of the two major carbohydrates fructose and glucose were determined by HPLC. The used honey sample consisted of 0.36 g glucose and 0.46 g fructose per gram honey, whereas agave syrup consisted of 0.19 g glucose and 0.7 g fructose/g (Table 1). Additional carbohydrates in honey and agave syrup, like the disaccharides maltose or sucrose were not determined via HPLC since they usually occur only in small quantities.

**Table 1:** Summary of the characterization of hereby used honey and agave syrup.

	**Honey**	**Agave syrup**
Glucose content (g/g)	0.36 ± 0.02	0.19 ± 0.01
Fructose content (g/g)	0.46 ± 0.04	0.70 ± 0.02
Water content (%)	17 ± 1	15 ± 1
Water activity	0.56	0.64
pH pure	3.4	3.9
pH diluted 1:10 (w/v)	3.61	4.29

The determined water content of both substrates was roughly the same, with 17% in honey and 15% in agave syrup. The thermodynamic water activity of honey is 0.56 and of agave syrup 0.64.

The pH of the measured samples was 3.4 for honey and 3.9 for agave syrup.

### Analysis of the synthesized products

**Figure 1:**
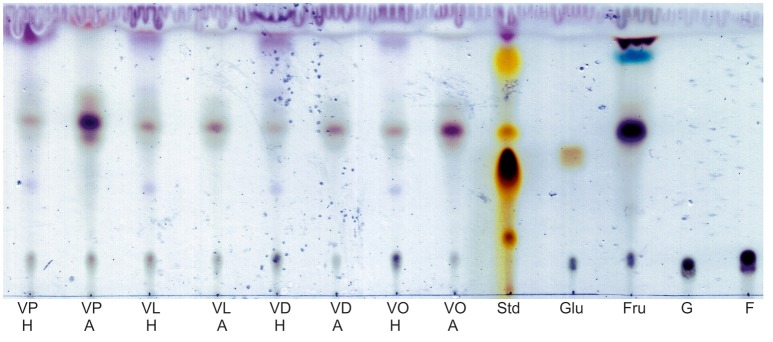
Visualization of synthesized glycolipids in honey and agave syrup after dying with an anisaldeyhde solution. Ten microliters of the extracts and four microliters of the standards were spotted on the TLC plate. VP, vinyl palmitate; VL, vinyl laurate; VD, vinyl decanoate; VO, vinyl octanoate; H, synthesis in honey; A, synthesis in agave syrup; Std, lab intern rhamnolipid standard; Glu, glucose based DES with VO; Fru, fructose based DES with VO; G, glucose, solved in an ethanol-water mixture; F, fructose in an ethanol-water mixture.

Extracted synthesis products of all reactions were visualized by TLC. After dyeing the TLC plates, several spots were visible indicating for several syntheses products. In comparison to the control reactions, the successful synthesis of sugar esters can be assumed. No major differences between the reactions in honey or agave syrup were observed (Figure 1).

The main spots of all extracts have a Rf (Table 2 retention factor) between 0.56 and 0.61, which increases slightly with the length of the used fatty acid vinyl ester. Further spots are visible in the extract with honey as substrate. With Rf-values are between 0.87 and 0.93, some of these spots can be located directly in the front of the mobile phase (VL H, VD H, andVO H). There are also very weak spots at Rf-values between 0.37 and 0.39.

**Table 2:** Rf-values of all visible spots and their corresponding possible compounds.

**Sugar source**	**Fatty acid este/ carbonyl chain**	**Main Rf-values**	**Possible compounds**
Honey	Vinyl palmitate/C14	0.93	Sugar-di- or poly-palmitate
		0.61	Fructose-mono-palmitate
		0.39	Unknown, occurs in negative controls, too
		0.12	Fructose
Agave syrup	Vinyl palmitate/C14	0.96	Sugar-di- or poly-palmitate
		0.68	Fructose-mono-palmitate
		0.60	Fructose-mono-palmitate
		0.55	Fructose- or glucose-mono-palmitate
		0.12	Fructose
Honey	Vinyl laurate/C12	0.90	Sugar-di- or poly-laurate
		0.58	Fructose-mono-laurate
		0.39	Unknown, occurs in negative controls, too
		0.12	Fructose
Agave syrup	Vinyl laurate/C12	0.58	Fructose-mono-laurate
		0.12	Fructose
Honey	Vinyl decanoate/C10	0.89	Sugar-di- or poly-decanoate
		0.57	Fructose-mono-decanoate
		0.38	Unknown, occurs in negative controls, too
		0.12	Fructose
Agave syrup	Vinyl decanoate/C10	0.57	Fructose-mono-decanoate
		0.12	Fructose
Honey	Vinyl octanoate/C8	0.87	Sugar-di- or poly-octanoate
		0.56	Fructose-mono-octanoate
		0.37	Unknown, occurs in negative controls, too
		0.12	Fructose
Agave syrup	Vinyl octanoate/C8	0.56	Fructose-mono-octanoate
		0.12	Fructose
DES glucose	Vinyl octanoate/C8	0.47	Glucose-mono-octanoate
		0.10	Glucose
DES fructose	Vinyl octanoate/C8	0.88	Sugar-di- or poly-octanoate
		0.83	Unknown, occurs in negative controls, too
		0.56	Fructose-mono-octanoate
		0.12	Fructose
Glucose Std		0.10	Glucose
Fructose Std		0.12	Fructose

With agave syrup as substrate, the vinyl palmitate extract showed some unique spots. There is one with a high Rf of 0.96 and a triple spot between Rf 0.55 and 0.68.

The visualization of extracted reaction products of glucose-based DES shows only one main spot, whereas the products of fructose-based DES shows two additional spots with higher Rf-values, of which the light blue one only occurs in this sample.

Additionally, every extract showed a spot on the same height as the sugar standards of glucose and fructose.

The lab internal rhamnolipid standard (Std) was used as a control for TLC separation and dyeing of the thin-layer plates.

### Determination of glycolipid masses via electrospray ionization quadrupole time-of-flight mass spectrometry (ESI-Q-ToF MS)

Purified sugar ester fractions were used for the validation of the successful enzymatic synthesis of glycolipids in honey and agave syrup, using vinyl octanoate as a representative substrate. The used fractions had a Rf-value of 0.55; the glucose- or fructose-octanoate had a calculated molar mass of 306.168 Da (M_G/F_). The presence of ions at m/z 289.312 (M_G/F_ − H_2_O + H^+^) and also the occurrence of the ammonium adduct at m/z 324.360 (M_G/F_ + ${\text{NH}}_{4}^{+}$) and the sodium adduct at m/z 329.311 (M_G/F_ + Na^+^) verifies the formation of glucose- or fructose-octanoate (M_G/F_), both in honey and agave syrup (Table 3 and Supplementary 3). Since glucose and fructose have the same molecular mass, the products cannot be easily distinguished via mass spectrometry alone.

**Table 3:** Observed m/z-values during ESI-Q-ToF experiments after flash purification of fraction 8 of with vinyl octanoate in honey, respective fraction 9 in agave syrup.

**Observed m/z-value**	**Calculated m/z-value**	**Sample/fraction**	**Corresponding fragment**
109.139	109.023	Honey + V-Oct/8	Sugar cleavage products
127.135	127.033	Honey + V-Oct/8	
145.158	145.043	Honey + V-Oct/8	
229.205		Honey + V-Oct/8	M_G/F_ − H_2_O − 2 CH_2_O + H^+^
271.290	271.145	Honey + V-Oct/8	M_G/F_ − 2 H_2_O + H^+^
289.318	289.158	Honey + V-Oct/8	M_G/F_ − H_2_O + H^+^
324.374	324.202	Honey + V-Oct/8	M_G/F_ + ${\text{NH}}_{4}^{+}$
329.336	329.158	Honey + V-Oct/8	M_G/F_ + Na^+^
109.139	109.023	Agave + V-Oct/9	Sugar cleavage products
127.135	127.033	Agave + V-Oct/9	
145.158	145.043	Agave + V-Oct/9	
189.196		Agave + V-Oct/9	Unknown
206.229		Agave + V-Oct/9	Unknown
224.234		Agave + V-Oct/9	Unknown
289.312	289.158	Agave + V-Oct/9	M_G/F_ − H_2_O + H^+^
324.360	324.202	Agave + V-Oct/9	M_G/F_ + ${\text{NH}}_{4}^{+}$
329.311	329.158	Agave + V-Oct/9	M_G/F_ + Na^+^
415.087	415.269	Honey/Agave unpurified	M2_G/F_ − H_2_O + H^+^
450.107	450.306	Honey/Agave unpurified	M2_G/F_ + ${\text{NH}}_{4}^{+}$

*Masses compare to glucose- or fructose-octanoate with a calculated molar mass of 306.168 Da (M_G/F_) and to glucose- or fructose-di-octanoate with 432.272 Da (M2_G/F_)*.

Several other identical masses were detected in both measured samples. They can be usually assigned to the glycolipid or to the free sugar species glucose or fructose and their cleavage products. Some other masses could not be clearly assigned to a certain substance.

In unpurified samples of a synthesis reaction in honey and in agave syrup, with vinyl octanoate, higher masses with m/z 415.087 and m/z 450.107 are occurring in addition to the above mentioned m/z-values. These values are matching to synthesized sugar-di-octanoates (M2_G/F_) with a calculated molar mass of 432.272. The observed m/z-values are M_2G/F_ − H_2_O + H^+^ and the ammonium adducts M_2G/F_ + ${\text{NH}}_{4}^{+}$.

By using other fatty acids, like the shorter vinyl hexanoate (278.14 Da) or longer vinyl laurate (362.23 Da), corresponding masses of formed sugar ester adducts were also detected (data not shown).

### Analysis of the synthesized products by NMR experiments

Mass spectrometry identified acylated sugars in purified synthesis products of both substrates. To distinguish between glucose- and fructose-octanoates, both with an identical calculated molar mass of 306.168 Da, purified and fractionated main components of a synthesis reaction in honey or agave syrup were analyzed by NMR spectroscopy. With honey as substrate, one clear major carbohydrate system was identified in the sample as glucose, starting from the anomeric protons of the ^1^H COSY and ^13^C HMBC spectra. Based on cross peaks of carbohydrate protons with lipid carbonyls in the ^1^H^13^C HMBC, the glucose moieties were acylated with octanoic acid at the C6 atoms (Table 4).

**Table 4:** Chemical shifts of the main product, presented in the purified, and fractionated sample of a synthesis reaction of vinyl octanoate in honey with an immobilized lipase.

**Glucose**	**C shift (ppm)**	**H shift (ppm)**
-C^1^H-O-	92.53	5.21
-C^2^H-	72.27	3.46
-C^3^H-	73.73	3.76
-C^4^H-	70.03	3.34
-C^5^H-	69.64	3.97
-C^6^H-(acylated C'174.13)	63.31	4.23
-C^6^'H-(acylated C'174.13)	63.31	4.35

*Despite purification via flash chromatography, the fractionated sample of the synthesis reaction in agave syrup show many impurities. Based on the identification of acetylated carbon atoms as C^1^H_2_ group, it is possible to state that it is fructose and not glucose*.

The purified synthesis product with agave syrup as substrate clearly revealed a cross peak between the lipid carbonyl C-atom and the CH_2_-carbohydrate group in the ^1^H^13^C HMBC spectra at 66.1 ppm (^13^C) and 4.22 and 4.11 ppm (^1^H) (Supplementary 4). This group shows no further cross peak in the 2D COSY. In the ^1^H^13^C HMBC it is connected to a quaternary carbon. This identifies the carbon atoms as C^1^H_2_ group of fructose, since glucose has no comparable group. A further assignment of the samples resonances was not possible due to spectral overlap with various impurities.

## Discussion

### Characterization of honey and agave syrup

For comparing and classifying the results, the herein used honey and agave syrup were characterized. The determined pH of 3.4 for honey coincides with other literature values. There it is often stated as pH 3.6, with variations between pH 3.3 and 7 (Wahdan, 1997; Kwakman et al., 2010). The pH in pure agave syrup was pH 3.9, and in the diluted solution it was pH 4.3, which is in the range of pH 4.1–5.5 for diluted agave syrup (Willems and Low, 2012).

The water content of honey and agave syrup were 17 and 15%, respectively, nearly the same, and the slight difference should only have a small impact on the synthesis reaction. The average water content for honey given in literature is 17.2% (Ajibola et al., 2012), for agave syrup it is 30% (Soto et al., 2011). However, it must be noted that agave syrup is often processed before it is used as natural sweetener, which explains the difference to the measured value.

Beckh et al. determined the water activity of 70 different types of honey (Beckh et al., 2004). Thirty-one were fluid with an a_w_ ranging from 0.52 to 0.64. The used honey for the synthesis of glycolipids has a water activity of 0.56, and is therefore in the a_w_ range for honey. The used agave syrup has an a_w_ of 0.64 and is near the value of 0.69 which Soto measured for agave syrup (Soto et al., 2011).

The most important value of both natural sugary products is their carbohydrate concentration. Both consist of over 80% of various sugars. In honey, the total amount of sugars varies around 80 and 83%, depending mostly on the pollen source and climate (Ajibola et al., 2012; United States Department of Agriculture, 2016). With 46% fructose and 36% glucose, the used honey reflects the average amount of these sugars in honey (40.9% fructose and 35.7% glucose). The used agave syrup has an 8% higher carbohydrate concentration compared to honey, divided in 70% fructose and 19% glucose. In a study from 2012, Willems et al. compare the major carbohydrates of 20 pure agave syrups. There, the mean value of fructose is 84.3 and 8.3% for glucose, which is similar to the measured data.

With a density of ~1.4 g/cm^3^, the volume of 2.5 ml of honey or agave syrup used for the synthesis reactions corresponds to a mass of 3.5 g of which over 2.8 g is sugar; this is equal to 15.5 mmol. Therefore, the applied amount of fatty acid vinyl esters (200 μl) is the limiting substrate in all reactions, because this amount corresponds, depending of the fatty acid vinyl ester, to a range between 0.6 and 1.03 mmol. In an earlier study by our group it was shown that the yield in a sugar-based DES under similar conditions is around 5%, which corresponds to a consumption of <1% of the used sugar (Siebenhaller et al., 2017). This suggests that the amount of honey or agave syrup used in this non-optimized process is in excess and therefore, does not limit sugar ester formation.

The characterization of the natural products was necessary in order to give general statement on the reproducibility of the reaction. Since all the parameters determined are correlating well with known literature values, it can be implied that our results are transferable to other honey and agave syrup samples.

### Thin-layer chromatography

**Figure 2:**
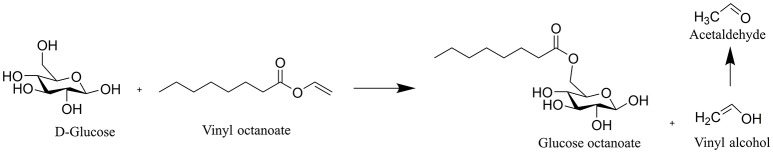
Lipase-catalyzed esterification between glucose and vinyl octanoate leads to the formation of glucose-octanoate and ethanol. The vinyl alcohol tautomerizes to acetaldehyde and evaporates, which prevents the back reaction. The same scheme applies to fructose and other fatty acid vinyl esters.

The extracts of all reactions in honey and agave syrup with the four tested fatty acid vinyl esters contained several compounds as visualized by positive spots on the dyed TLC plate (Figure 1). To verify that these spots were formed by the enzymatic synthesis (Figure 2), negative controls without enzyme were compared with the synthesis reactions (Supplementary 1, 2). Thereby, it was clearly shown that without the addition of enzyme formation of glycolipid is not possible.

When comparing the synthesis products in honey and agave syrup with the fructose-based DES and vinyl octanoate as substrate, the deduced product spots have the same Rf-values (0.56 and 0.12, respectively, Table 2). However, the synthesis product of the glucose-based DES has a lower Rf of 0.47, which can not be seen in any other synthesis reaction. This may indicate that fructose might be more favored by the used lipase rather than glucose. Furthermore, in all reaction extracts a spot at Rf 0.12 is visible, corresponding to free fructose or glucose, due to the similar running characteristics of fructose and glucose.

On the TLC plate it is possible to see a small difference in the Rf-values of the spots, depending on the length of the carbonyl chains. When using vinyl palmitate, the substrate with the longest carbonyl chain, the main spot has a Rf of 0.61, and with vinyl octanoate it slightly decreases to 0.56. The height of the spots depends on the length of the fatty acid chain, which is linked with an ester bond to the sugar.

When using honey, additional spots with Rf-values between 0.87 and 0.93 are visible. Some of those can also be located directly in the front of the mobile phase (VL H, VD H, and VO H). These indicates the formation of di- or polyacylated sugars, which also have been observed several times in prior experiments (Siebenhaller et al., 2016).

With agave syrup and vinyl palmitate as substrates, the synthesis extract shows some unique spots: One with a high Rf of 0.96, which also indicates for di- or polyacylated sugars. Furthermore, the same extract shows a triple spot between Rf 0.55 and 0.68. We hypothesize that the formed sugar esters might have several configurations, leading to a slightly different polarity, resulting in a different running behavior. In the other reactions this triple spot is not clearly visible.

By comparing the control and synthesis reactions, it is noticeable that glycolipids only appear in the synthesis reactions. In the samples of the fructose based DES a blue spot at Rf 0.83 occures in all reactions, including the negative controls. Therefore, it is no synthesis product but might be a pH-depending configuration of the fructose molecule, since it does not occur in the pure fructose control.

### Identification of the synthesized sugar esters

It was possible to detect the masses of glucose- and fructose-octanoate via mass spectrometry of purified samples. The detected m/z-values of 289.31, 324.36, and 329.32 indicate the presence of the predicted sugar esters (306.168 Da), synthesized in honey and agave syrup. Due to the identical mass of glucose- and fructose-octanoate, it cannot be conclusively clarified which sugar is connected to the octanoate via mass spectrometry. The NMR data confirm the formation of sugar esters in honey and agave syrup. It was determined that the synthesized product in honey is glucose-6-octanoate. This indicates that the used iCalB seems to prefer to acylate glucose at the C6, which was already shown (Siebenhaller et al., 2017). Whether fructose-octanoate is also a product of the synthesis reaction in honey cannot conclusively be clarified, as it may have been removed from the measured sample during the purification step. Vice versa, in the purified synthesized product sample in agave syrup, only acylated fructose was detected. This sample shows various impurities; therefore, further statements should only be taken with caution.

The formation of sugar-di-octanoates was proven in unpurified samples by the occurrence of the sodium adduct. This is consistent with previous results of synthesis reactions in DESs, in which the formation of mono- and di-acylated sugars were observed via ESI-Q-ToF-MS, too (Siebenhaller et al., 2017). Due to their separation during flash chromatography, sugar-di-octanoates were only detected in the unpurified synthesis extracts. Since the yield of di-esters seems very low, NMR analysis was not possible.

The smaller observed masses (m/z 109.139, 127.135, and 145.158) did not correlate to a formed sugar ester. Therefore, further MS experiments were carried out with pure glucose in which these masses also occurred, indicating for sugar cleavage products.

The m/z 229.205, which occurs in the honey fraction, can be explained by the loss of two CH_2_O molecules and a water cleavage from the formed sugar-octanoate (Lie et al., 2015).

Hitherto, the three detected masses between m/z 189.196 and m/z 224.234 cannot be assigned to an expected product. These masses could, however, match one of the other ingredients of agave syrup.

By synthesis in honey, with a ratio of fructose to glucose of 1:0.78, only glucose-octanoate was detected. In agave syrup the ratio is 1:0.26; after purification of the synthesized products only fructose-octanoate was identified. This may indicate that our first presumption was wrong, and the used iCalB prefers glucose to fructose at similar sugar ratios. Whether this is correct or caused by other factors like temperature, fatty acid length or other components in the honey or agave syrup influence the substrate preference, has to be further investigated.

## Conclusion

It was shown that ordinary honey and, as vegan alternative, agave syrup are suitable to act simultaneously as substrates and solvents for the enzymatic synthesis of sugar esters. The successful formation of the sugar esters was proved by TLC, MS and multidimensional NMR experiments, which were compared to known reactions in sugar-based DESs.

The produced sugar esters can potentially be used in the pharmaceutical or cosmetics industry to improve existing products or to replace conventional surfactants. But to do so, important attributes and dermatological properties have to be determined.

Important characteristics like the content of the main sugars, water activity and water content as well as the pH of the herein used honey and agave syrup were determined and compared to literature data. However, this have to be further investigated since they might have an influence on the reaction, yields, and products.

The used process is not yet optimized, but it demonstrated that the reaction system on which this work is based is very versatile and stable. Furthermore, it shows that the used lipase can overcome the used unconventional media and is able to reverse their hydrolytic activity and synthesize sugar esters despite the high water content and water activity. This result may open the door for other natural substrates like maple syrup, rice syrup or the commercially important corn syrup.

## Author contributions

SS: performance of experiments, conception of the work, and supervise JG. Write the main part of the manuscript. JG: Substantial performance of all experiments and revising of the final version. AI: performed the pH measurements and critical revision of the manuscript. CM-G: execution and evaluation of the NMR analysis. FK and GB-W: performed the ESI-Q-ToF-MS measurements, writing parts of the MS-section, and critically revising the manuscript. KO and CS: critical revision of the work and manuscript and important intellectual content.

### Conflict of interest statement

The authors declare that the research was conducted in the absence of any commercial or financial relationships that could be construed as a potential conflict of interest.

## References

[B1] Ajibola A. Chamunorwa J. P. Erlwanger K. H. (2012). Nutraceutical values of natural honey and its contribution to human health and wealth. Nutr. Metab. 9:61. 10.1186/1743-7075-9-61 22716101 PMC3583289

[B2] Azeredo L. D. C. Azeredo M. A. A. De Souza S. R. Dutra V. M. L. (2003). Protein contents and physicochemical properties in honey samples of *Apis mellifera* of different floral origins. Food Chem. 80, 249–254. 10.1016/S0308-8146(02)00261-3

[B3] Beckh G. Wessel P. Lüllmann C. (2004). Natürliche Bestandteile des Honigs: Hefen und deren Stoffwechselprodukte–Teil 2: Der Wassergehalt und die Wasseraktivität als Qualitätsparameter mit Bezug zum Hefewachstum. Deuts. Leben. Rundschau 100, 14–17.

[B4] Burlando B. Cornara L. (2013). Honey in dermatology and skin care: a review. J. Cosmet. Dermatol. 12, 306–313. 10.1111/jocd.12058 24305429

[B5] Cao L. Bornscheuer U. T. Schmid R. D. (1999). Lipase-catalyzed solid-phase synthesis of sugar esters. influence of immobilization on productivity and stability of the enzyme. J. Mol. Catal. B Enzym. 6, 279–285. 10.1016/S1381-1177(98)00083-6

[B6] Coulon D. Ismail A. Girardin M. Rovel B. Ghoul M. (1996). Effect of different biochemical parameters on the enzymatic synthesis of fructose oleate. J. Biotechnol. 51, 115–121. 10.1016/0168-1656(96)01588-X

[B7] Crane E. (1980). A Book of Honey. Oxford University Press.

[B8] Degn P. Pedersen L. H. Duus J. Ø. Zimmermann W. (1999). Lipase-catalysed synthesis of glucose fatty acid esters in tert -butanol. Biotechnol. Lett. 21, 275–280. 10.1023/A:1005439801354

[B9] Dörsam S. Fesseler J. Gorte O. Hahn T. Zibek S. Syldatk C.. (2017). Sustainable carbon sources for microbial organic acid production with filamentous fungi. Biotechnol. Biofuels 10:242. 10.1186/s13068-017-0930-x 29075326 PMC5651581

[B10] Ducret A. Giroux A. Trani M. Lortie R. (1996). Characterization of enzymatically prepared biosurfactants. J. Am. Oil Chem. Soc. 73, 109–113. 10.1007/BF02523456

[B11] Enthart A. Freudenberger J. C. Furrer J. Kessler H. Luy B. (2008). The CLIP/CLAP-HSQC: pure absorptive spectra for the measurement of one-bond couplings. J. Magn. Reson. 192, 314–322. 10.1016/j.jmr.2008.03.009 18411067

[B12] Gheldof N. Wang X. H. Engeseth N. J. (2002). Identification and quantification of antioxidant components of honeys from various floral sources. J. Agric. Food Chem. 50, 5870–5877. 10.1021/jf0256135 12358452

[B13] Jiménez M. M. Fresno M. J. Sellés E. (1994). The galenic behaviour of a dermopharmaceutical excipient containing honey. Int. J. Cosmet. Sci. 16, 211–226. 10.1111/j.1467-2494.1994.tb00098.x 19250489

[B14] Kim S. H. Park S. Yu H. Kim J. H. Kim H. J. Yang Y.-H. (2016). Effect of deep eutectic solvent mixtures on lipase activity and stability. J. Mol. Catal. B Enzym. 128, 65–72. 10.1016/j.molcatb.2016.03.012

[B15] Kwakman P. H. te Velde A. A. de Boer L. Speijer D. Vandenbroucke-Grauls C. M. Zaat S. A. (2010). How honey kills bacteria. FASEB J. 24, 2576–2582. 10.1096/fj.09-150789 20228250

[B16] Lie A. Stensballe A. Pedersen L. H. (2015). Structural analyses of sucrose laurate regioisomers by mass spectrometry techniques. J. Carbohydr. Chem. 34, 206–214. 10.1080/07328303.2015.1021475

[B17] Liu P. Hao J.-W. Mo L.-P. Zhang Z.-H. (2015). Recent advances in the application of deep eutectic solvents as sustainable media as well as catalysts in organic reactions. RSC Adv. 5, 48675–48704. 10.1039/C5RA05746A

[B18] Ohmenhaeuser M. Monakhova Y. B. Kuballa T. Lachenmeier D. W. (2013). Qualitative and quantitative control of honeys using NMR spectroscopy and chemometrics. ISRN Anal. Chem. 2013, 1–9. 10.1155/2013/825318

[B19] Pöhnlein M. Ulrich J. Kirschhöfer F. Nusser M. Muhle-Goll C. Kannengiesser B. (2015). Lipase-catalyzed synthesis of glucose-6-O-hexanoate in deep eutectic solvents. Eur. J. Lipid Sci. Technol. 117, 161–166. 10.1002/ejlt.201400459

[B20] Šabeder S. Habulin M. Knez Ž. (2006). Lipase-catalyzed synthesis of fatty acid fructose esters. J. Food Eng. 77, 880–886. 10.1016/j.jfoodeng.2005.08.016

[B21] Siebenhaller S. Hajek T. Muhle-Goll C. Himmelsbach M. Luy B. Kirschhöfer F.. (2017). Beechwood carbohydrates for enzymatic synthesis of sustainable glycolipids. Bioresour. Bioprocess 4:25. 10.1186/s40643-017-0155-7 28680800 PMC5487819

[B22] Siebenhaller S. Muhle-Goll C. Luy B. Kirschhöfer F. Brenner-Weiss G. Hiller E. (2016). Sustainable enzymatic synthesis of glycolipids in a deep eutectic solvent system. J. Mol. Catal. B Enzym. 133, S281–S287. 10.1016/j.molcatb.2017.01.015

[B23] Soto J. L. M. González J. V. Nicanor A. B. Ramírez E. G. R. (2011). Enzymatic production of high fructose syrup from *Agave tequilana fructans* and its physicochemical characterization. Afr. J. Biotechnol. 10, 19137–19143. 10.5897/AJB11.2704

[B24] Tarahomjoo S. Alemzadeh I. (2003). Surfactant production by an enzymatic method. Enzyme Microb. Technol. 33, 33–37. 10.1016/S0141-0229(03)00085-1

[B25] United States Department of Agriculture (2016). National Nutrient Database for Standard Reference.

[B26] Viuda-Martos M. Ruiz-Navajas Y. Fernández-López J. Perez-Álvarez J. A. (2008). Functional properties of honey, propolis, and royal jelly. J. Food Sci. 73, 117–124. 10.1111/j.1750-3841.2008.00966.x 19021816

[B27] Wahdan H. A. (1997). Causes of the antimicrobial activity of honey. Infection 26, 26–31. 10.1007/BF02768748 9505176

[B28] Watanabe T. Katayama S. Matsubara M. Honda Y. Kuwahara M. (2000). Antibacterial carbohydrate monoesters suppressing cell growth of *Streptococcus mutans* in the presence of sucrose. Curr. Microbiol. 41, 210–213. 10.1007/s002840010121 10915210

[B29] White J. W. Doner L. W. (1980). Honey composition and properties. Beekeep. U.S. Agric. Hand B 335, 82–91.

[B30] Willems J. L. Low N. H. (2012). Major carbohydrate, polyol, and oligosaccharide profiles of agave syrup. application of this data to authenticity analysis. J. Agric. Food Chem. 60, 8745–8754. 10.1021/jf3027342 22909406

[B31] Yan Y. Bornscheuer U. T. Cao L. Schmid R. D. (1999). Lipase-catalyzed solid-phase synthesis of sugar fatty acid esters. Enzyme Microb. Technol. 25, 725–728. 10.1016/S0141-0229(99)00106-4

