# Supplementary figures and images for: Lipase-Catalyzed Synthesis of Sugar Esters in Honey and Agave Syrup
Source: Front Chem. 2018 Feb 12;6:24. doi: 10.3389/fchem.2018.00024 (PMC5816588; doi:10.3389/fchem.2018.00024)

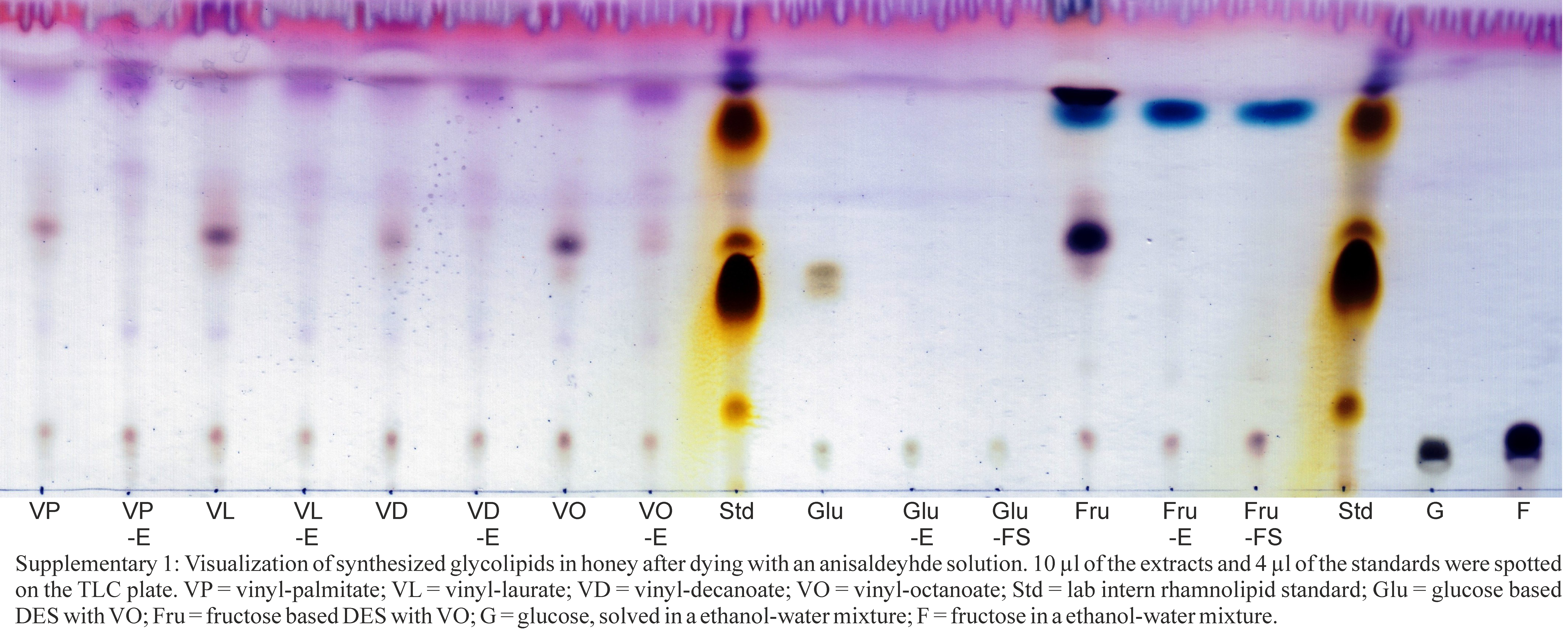

Supplement: Supplementary file 1 [file Image1.tif]

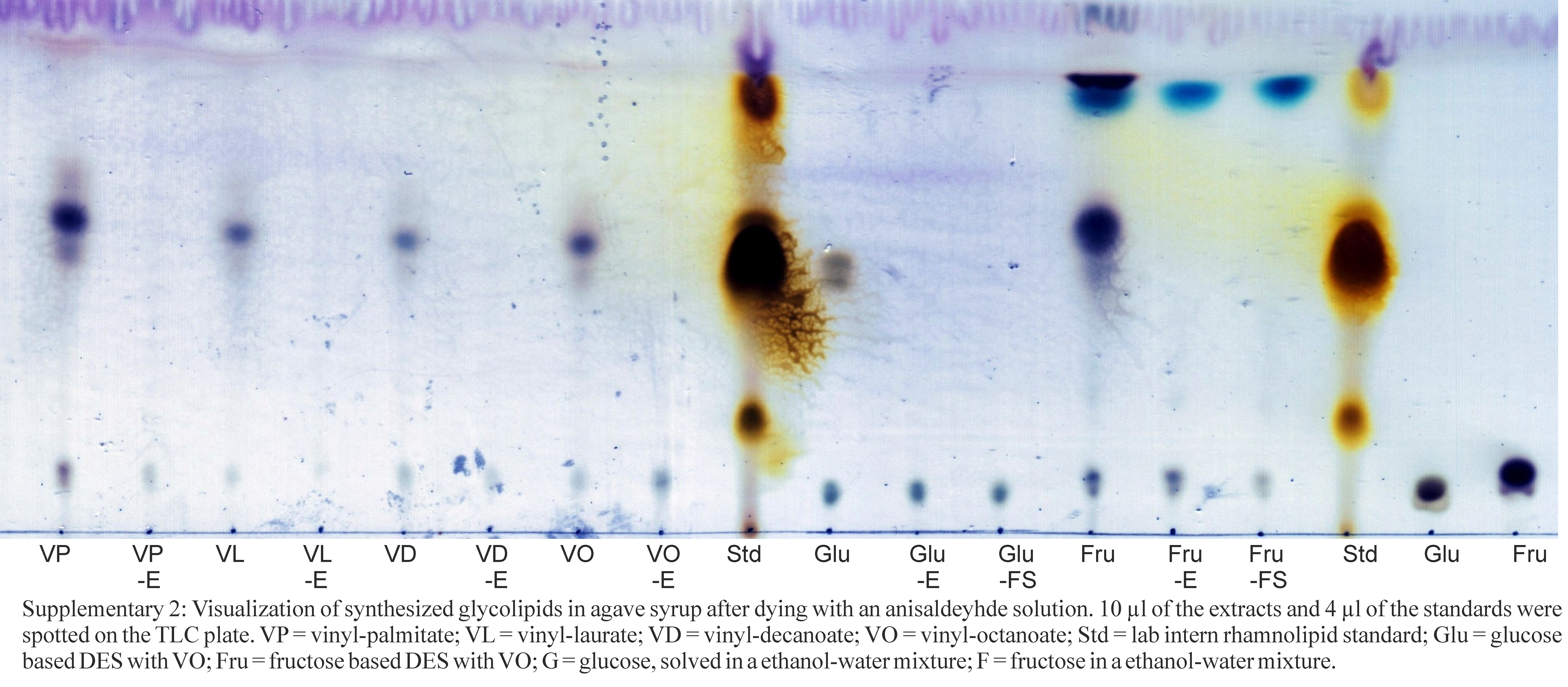

Supplement: Supplementary file 2 [file Image2.tif]
