# Supplementary material for: Lipase-Catalyzed Synthesis of Sugar Esters in Honey and Agave Syrup
Source: Front Chem. 2018 Feb 12;6:24. doi: 10.3389/fchem.2018.00024 (PMC5816588; doi:10.3389/fchem.2018.00024)
Supplement: Supplementary file 3 [file Image3.pdf]

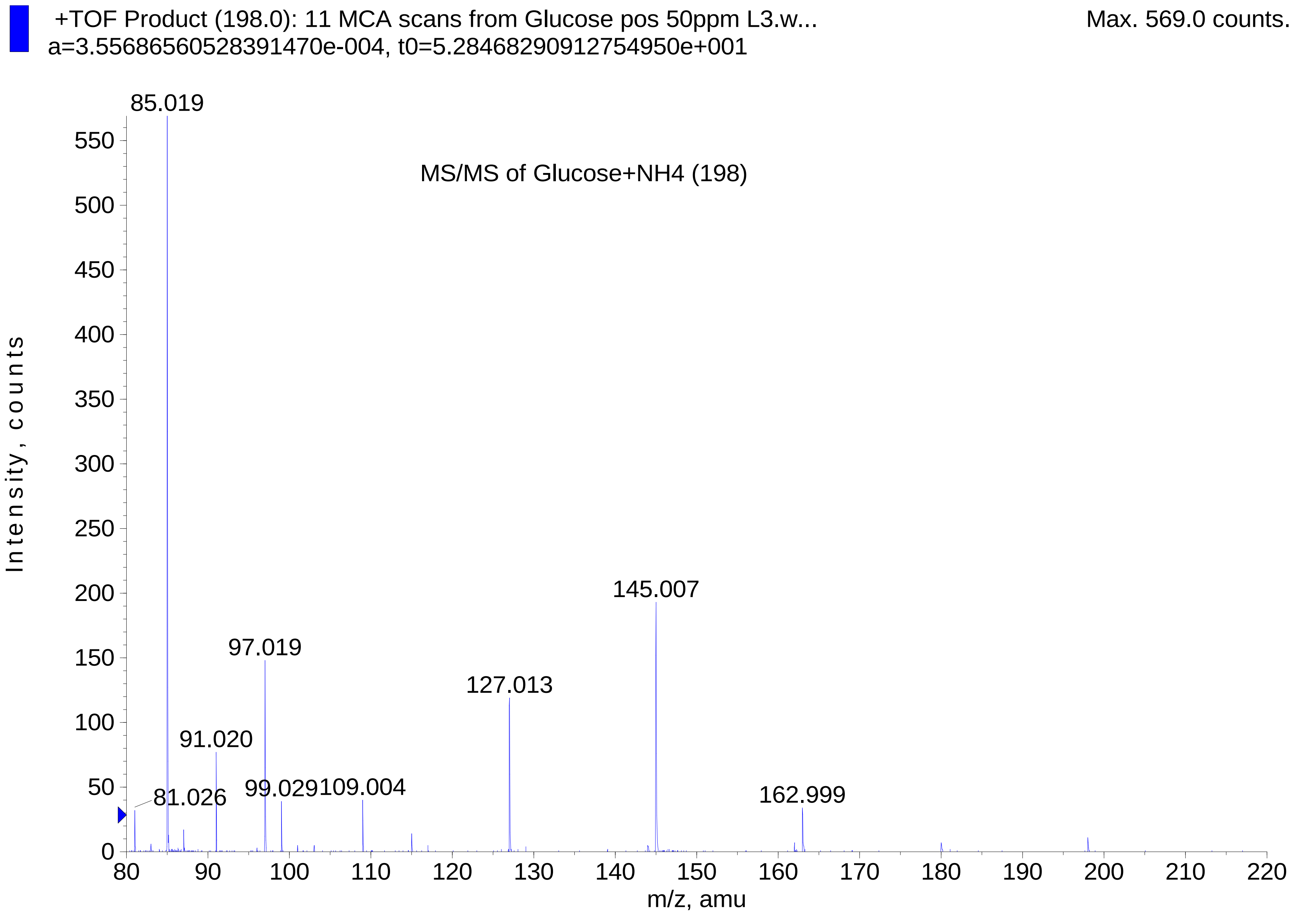

Supplementary 3: Verification of the sugar cleavage products

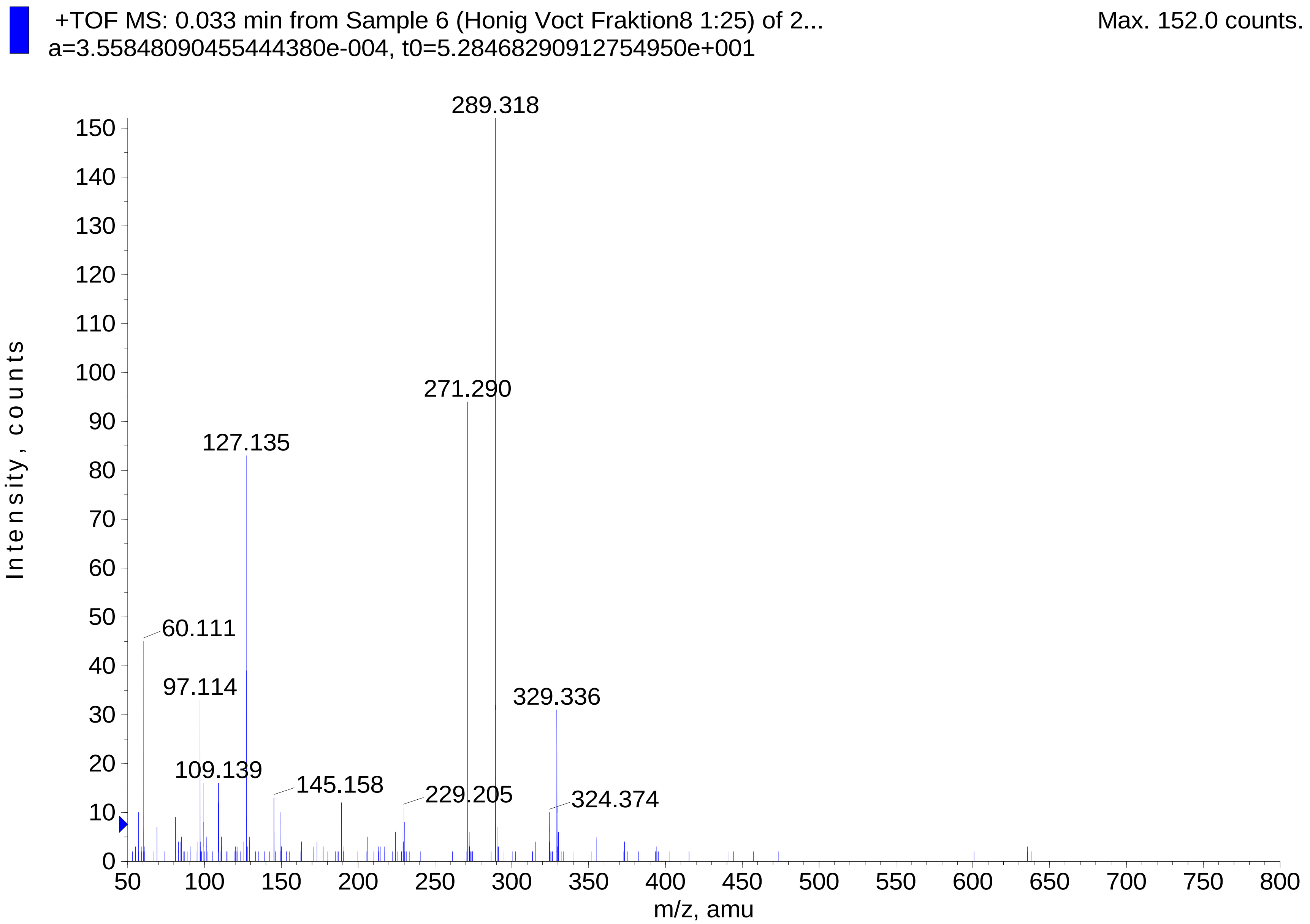

Synthesis with honey and vinyl octanoate, fraction 8

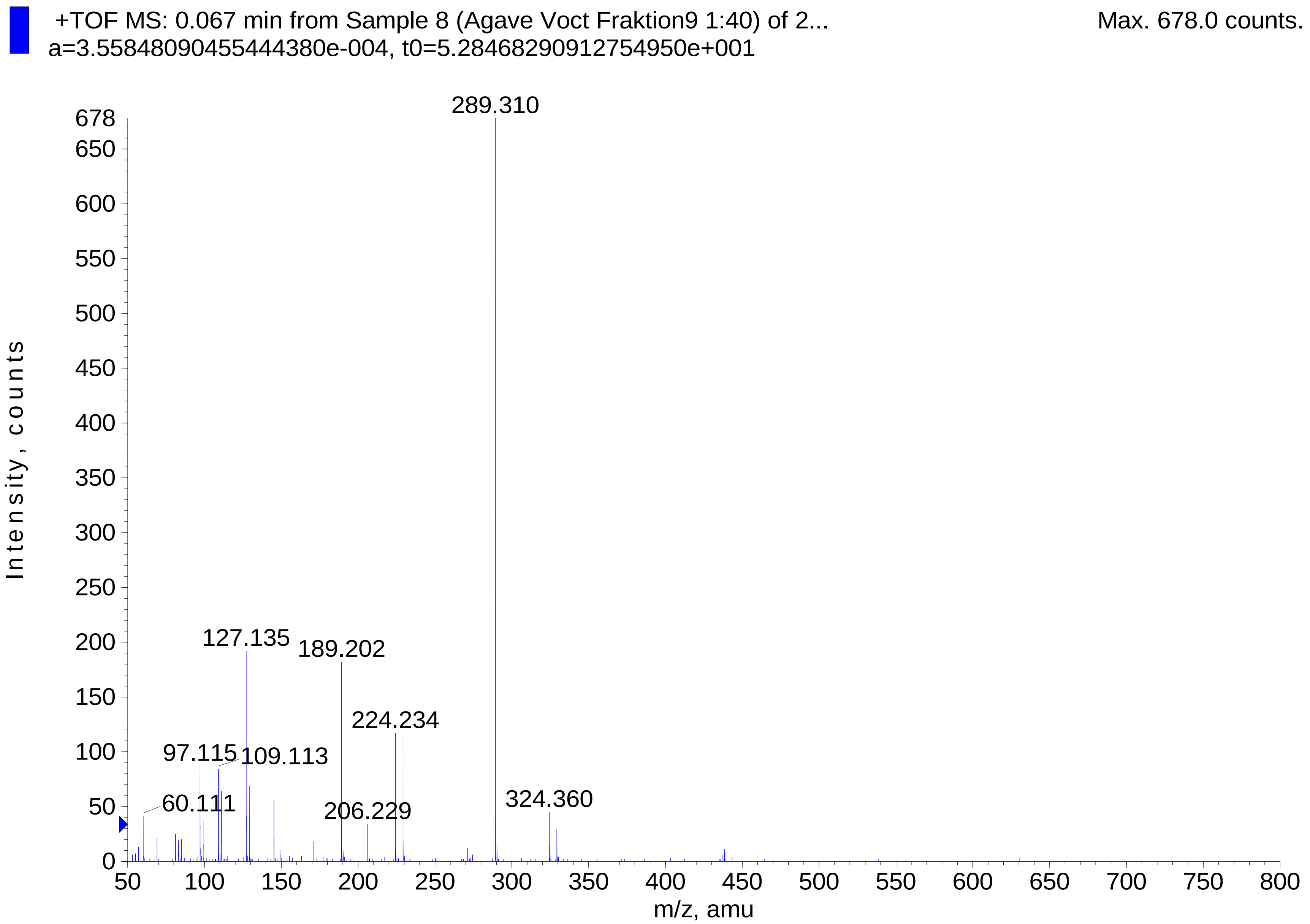

Synthesis with agave syrup and vinyl octanoate, fraction 9

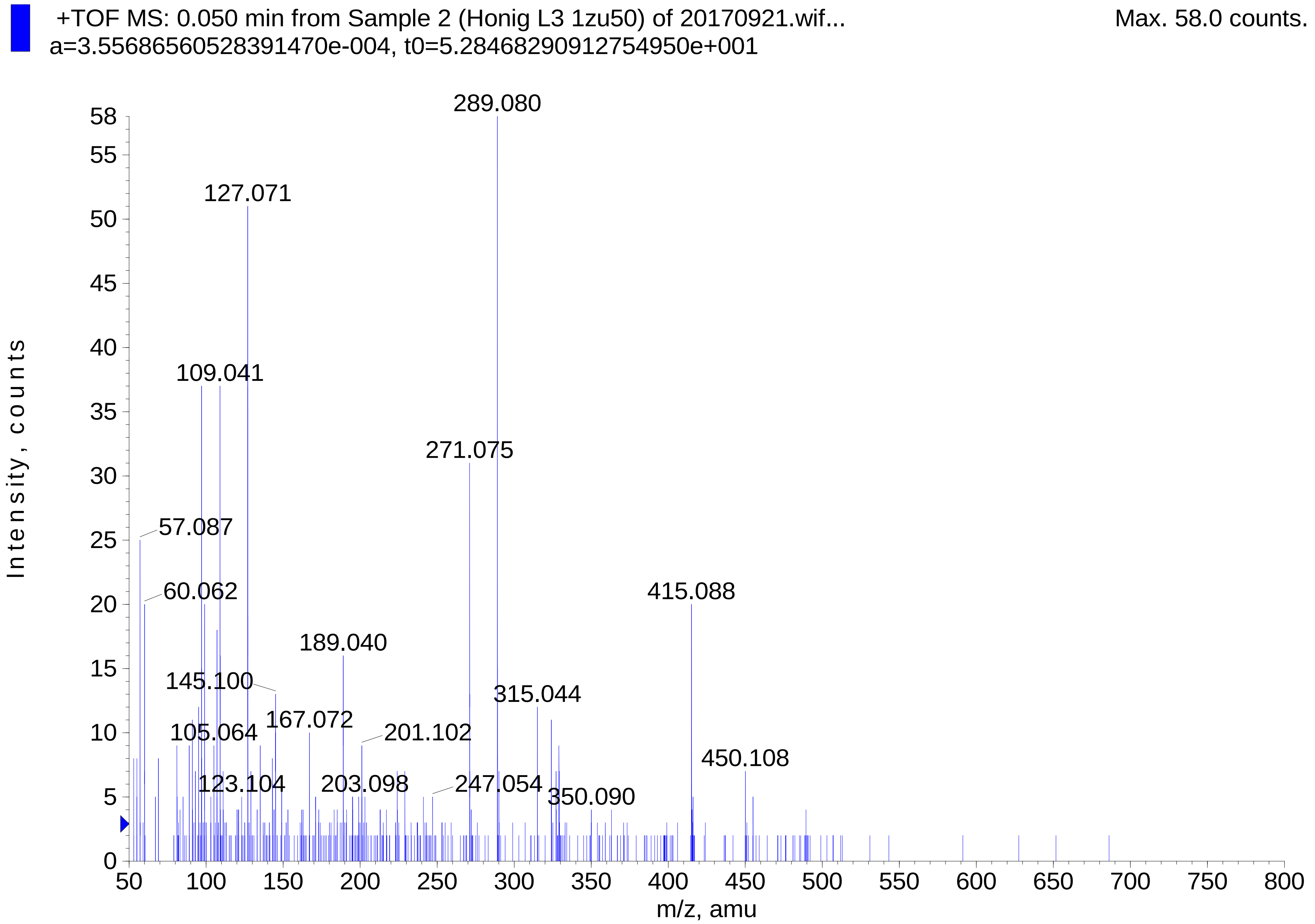

Synthesis with honey and vinyl octanoate, unpurified

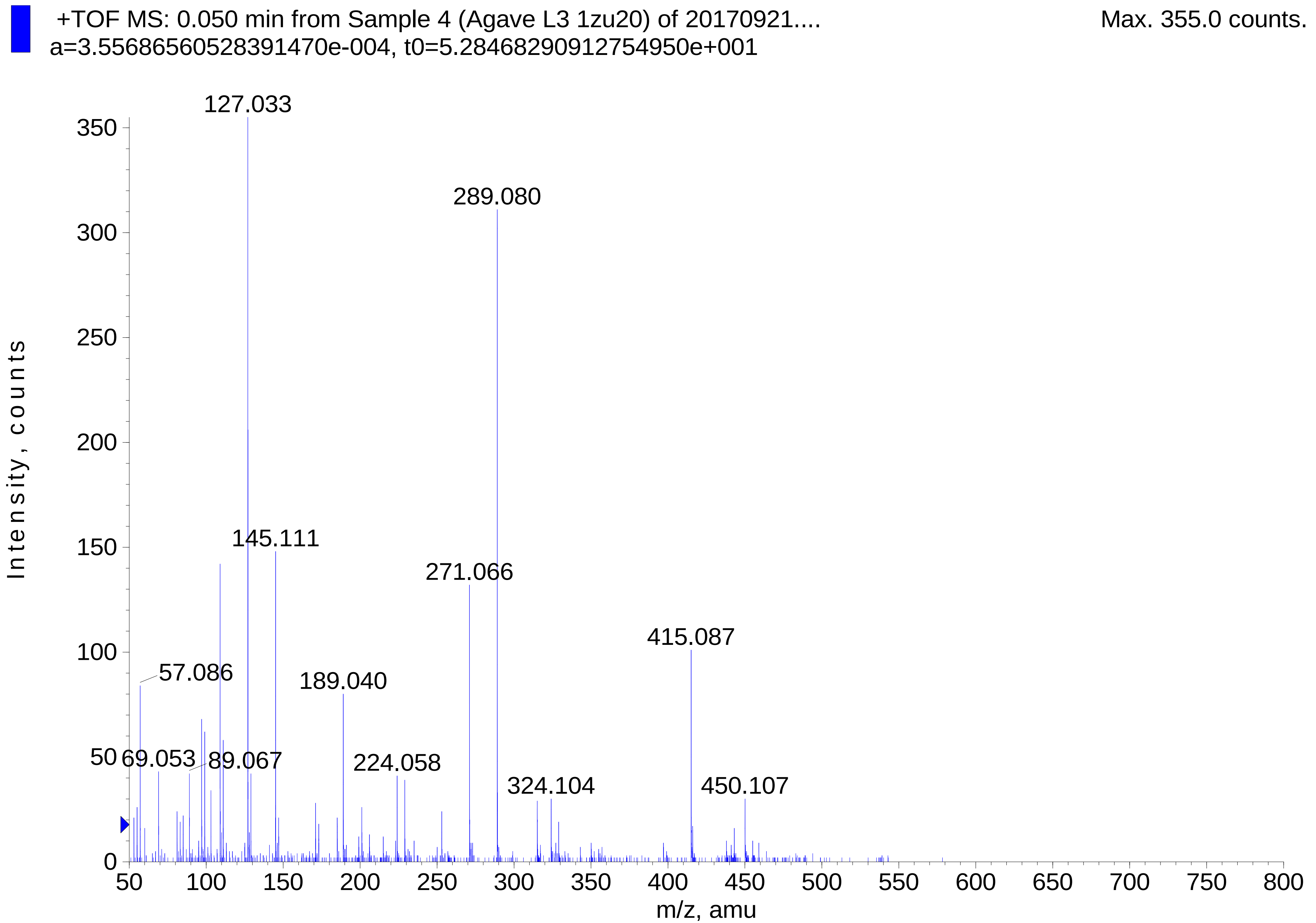

Synthesis with agave syrup and vinyl octanoate, unpurified
